# Working short and working long: can primary healthcare be protected as a public good in Lebanon today?

**DOI:** 10.1186/s13031-021-00359-4

**Published:** 2021-04-07

**Authors:** Randa Sami Hamadeh, Ola Kdouh, Rawan Hammoud, Enrica Leresche, Jennifer Leaning

**Affiliations:** 1 Lebanese Ministry of Public Health, Beirut, Lebanon; 2 Global Health Team of Experts, Beirut, Lebanon; 3 London School of Hygiene and Tropical Medicine, London, UK; 4 Harvard François Xavier Bagnoud Center for Health and Human Rights, Boston, USA

**Keywords:** Primary health care, Covid-19, Protracted conflict, Lebanon, Health system resilience, Disaster response

## Abstract

In this commentary we propose four questions to be addressed while building a meaningful public primary healthcare response in Lebanon today. These questions emerge from two imperatives: the necessity to consider both short- and longer-term struggles in a context of protracted conflict and the need to protect public health as a public good whilst the public Primary Healthcare Network (PHCN) is facing the Covid19 pandemic. In order to identify how these questions are related to the need to be working short and long, we look at the imprints left by past and present shocks. Profound shocks of the past include the Lebanese civil war and the Syrian refugee crisis. We analyse how these shocks have resulted in the PHCN developing resilience mechanisms in order to ensure a space for healthcare provision that stands public in Lebanon today. Then, we consider how two present shocks -- the economic breakdown and the blast of ammonium nitrate in Beirut port -- are affecting and threatening the progress made by the PHCN to ensure that primary healthcare remains a public good, a fragile space acquired with difficulty in the past half century. We identify what questions emerge from the combined consequences of such traumas, when the immediate constraints of the present meet the impediments of the past. We consider what such questions mean more broadly, for the people living in Lebanon today, and for the PHCN ability to respond to the Covid 19 pandemic in a relevant way. Our hypothesis is that in a protracted conflict, such as the one defining the circumstances of Lebanon now, public access to primary healthcare might persist for the people as one safeguard, in which social and moral continuity can be anchored to protect a sense of public good.

## Background

Protracted conflicts arise from multiples of existential shocks—active armed conflicts (non-international or international), social strife resulting from poorly resolved past conflicts, governmental collapse, system-wide economic failure, hostile invasions, mass refugee influx, massively destructive disasters, large-scale internal displacement occasioned by local conflict or climate-driven assaults on livelihoods, and, rarely, pandemics. These shocks may occur sequentially or at times accumulate in a short time frame, often because they may be related to or may increase the negative dynamics of previous calamities. The humanitarian community is only just beginning to recognize these complexities and their implications for relief and development. These questions were recently examined (by the International Committee of the Red Cross) to identify key characteristics of any protracted conflict [1]. All of these key characteristics are active in the case of Lebanon [2–4]. A protracted conflict claims longevity, either as one that persists for decades or one that merges and entangles with others that develop within one country. A protracted conflict fragments and mutates, engaging new armed groups, political factions, disruptions, and forces a re-framing of priorities for positive intervention. It arises episodically, with spikes of distress or violence that are not of equal intensity. It is internationalized, prompting other States to intervene in various forms. It imposes a cumulative impact, wherein infrastructure is degraded, utilities and services decay, social capital erodes, and living conditions decline. It induces pervasive fragility of governance and reduced capacity to withstand economic and political shocks or massive disasters. And over time it intensifies the suffering of its people, who move to escape one shock and then another, resulting in a rising number of impoverished people moving within or beyond national borders.

The interactive relationship between protracted conflicts and public health has been described in the past decades, including the direct and indirect consequences that conflicts impose on health, as well as the notion of “breakdown” of societies where civilians bear the heaviest part of the burden [1, 5, 6]. In protracted conflicts the efforts undertaken necessitate dealing with broken human receptive capacities, at a pace that requires stemming the worst impact of the immediate disasters, when many aspects of the society have been damaged for a long time. In this paper, the simultaneous focus on meeting immediate needs and on alleviating the deep cumulative impact is referred to as the necessity to be “working short and working long” [1]. From this perspective, the role of a public health system can rapidly become compromised but also may persist as an important beacon of hope and of an aspirational value given to human life. The peer-reviewed literature on this topic is scant but includes insights captured in accounts of humanitarians and human rights observers in wars and crises, as well as in historical accounts of protracted conflicts (including the many post-colonial wars) arising in the decades after World War II.

One major constraint of the literature on this subject, however, is the difficulty of measuring events as they happen, forcing a reliance on retrospective accounts. Real time questions related to the public health decisions that have to be made as a deep multi-faceted crisis unfolds can be explored in Lebanon today [2, 4]. These questions relate to the need to understand the trade-offs embedded in any decisions taken to respond to Covid-19 as the acute pandemic spreads while resource constraints continue to tighten for essential public health services such as vaccinations, maternal and child healthcare, and support to those with non-communicable disease. This understanding is needed to discern how these trade-offs are negotiated for both the short and long term. Such negotiations will depend on prior resilience mechanisms, how these might be disrupted by the current crisis and how such mechanisms can be protected. Understanding the inherited fractures of the past allows us to uncover the cumulative impact in a population affected by different layers of crises; to identify which essential health needs might be at risk and for whom; to discern how the short and long term responses can possibly include the most vulnerable in both phases; and to address the ethical questions embedded in choices that must be made in a population-based response [1]. This complex task now confronts those delivering public primary healthcare (PHC) in Lebanon. Their approach, strategies, and compromises are analysed in this commentary.

## Working long and working short

In this section we document the main past shocks that the Lebanese primary health care network (PHCN) has been dealing with since 1975. Then, we analyse how this network has been frayed by present shocks. Finally, we consider how the combination of both shocks confront the public health system with significant trade-offs in crafting an appropriate response to the Covid-19 crisis pandemic--when the economy is collapsing, and the Beirut port and half the city lie in ruins.

### The scars of the past

Historically, the country’s autonomy has been periodically threatened by regional and international nation-states [3, 7]. The pluralistic society of Lebanon has managed to maintain a relative equilibrium in the midst of such turbulent times. However, profound shocks such as the Lebanese civil war (1975–1990) and the Syrian crisis (2011) have contributed to shape the Lebanese healthcare system in a very peculiar way. We describe some of these characteristics below.

First, the shocks of the past have shaped a social organisation of healthcare along sectarian lines [3, 7, 8]. Following the independence of the country in 1943, the National Pact imposed a sectarian distribution of governmental power along the lines initiated during the French mandate [7]. At the end of the Lebanese civil war in 1990, the country’s governance structure recapitulated the same sectarian divisions in the Taef Agreement [7]. This distribution of political powers has become over the years increasingly dysfunctional and kleptocratic [3, 8]. Post-civil war reforms were proposed by the MoPH to promote health and to protect the population based on the Alma Ata Declaration [7]. Such reforms embodied the attempt to maintain a public space in which to resist the growth of the private and for profit healthcare provision flourishing after the civil war [7]. As these reforms struggled to take root in the midst of political sectarian turmoil and repeated economic recessions, the funds allocated to the public health system progressively decreased [7].

Second, the civil war forced the Ministry of Public Health (MoPH) to rely on disparate non-state actors and private providers [2, 4, 7–9]. In its post-war weakened capacity, the MoPH had to accommodate to a hospital-centred and specialized provision of care, under the influence of the private sector [2, 4, 9]. This long-standing adjustment has left the MoPH with meagre financial means, insufficient human resources, and little authority to guide and coordinate the provision of public healthcare. Between 1998 and 2007, 25% of the MoPH staff who retired were not replaced [7]. Due to conflicting sectarian and private interests, the law to provide for one single pool of funds to cover the entire Lebanese population has never been passed [9]. Primary healthcare has received very little funding within the MoPH budget: It is estimated that 5% is allocated to prevention and primary healthcare services while 79% is used to reimburse contracted hospital services [7]. These disrupted efforts to establish an integrated approach have shaped fragmented sub-systems acting through separate mechanisms to provide primary healthcare services to various populations of interest.

Third, the shocks as they unfolded over the past decades have progressively excluded a growing number of groups of people from the costly for-profit provision of healthcare [4]. The MoPH is the funder of last resort for the Lebanese (over 50% of whom lack any forms of insurance) resulting in a heavy drain on public funds to cover service contracts with private hospitals [2, 4, 9]. While national insurance schemes might cover predefined outpatient services on a reimbursement basis for Lebanese employed in the formal sector, most outpatient services are financed by out-of-pocket (OOP) payments for people not included in such schemes. The result is that vulnerable Lebanese patients encounter financial barriers to seeking any form of primary healthcare [7]. The chronically impoverished Palestinian refugees rely on the 70 plus years of support from the United Nations Relief and Works Agency (UNRWA) covering most of their essential health needs since 1948 [2]. The influx of over one million Syrian refugees into Lebanon increased the population by over 30%, adding another group of people unable to afford the expense of private healthcare [4, 10]. Based on its hard experience with Palestinian camps, the Lebanese government set up a de facto “no-camp” policy for Syrian refugees, which resulted in placing refugees in communities or existing systems which were already weakened or failing [4, 9]. This strategy meant that a population of over one million impoverished refugees had to be incorporated into a system of care that was already struggling to meet the needs of previously impoverished populations [2, 4]. As a result, the percentage of Syrian refugees in the PHC network increased from 12% in 2012 to 46% in 2018, an illustration of the impact of the Syrian crisis on the provision of services at the primary level of care.

Fourth, the Syrian crisis also brought an additional influx of non-governmental organizations (NGOs) and donors, resulting in further fragmentation of the systems of care [4]. This increasingly complex network created overlaps for patients as well as providers [4, 9, 11]. The growing number and variety of players shaped the primary healthcare provision into an even more complex array of intermingled actors over the years. As these different shocks have unfolded, the provision of public healthcare has been squeezed into an underfunded and fragmented set of arrangements for those too poor to have access to the privatized for-profit providers of primary healthcare [4].

Fifth, the past shocks allowed important geographic disparities to surface. Geographic disparities include underserved areas along the borders which have been traditionally inhabited by poor Lebanese and Syrian seasonal workers [4, 12] and urban areas where a range of impoverished groups (poor Lebanese, Palestinian refugees, Syrian refugees) may have access to subsidized services [11]. The arrival of Syrian refugees imposed a drastic increase in the number of people to be accommodated in the most underserved areas of the system [4, 9]. To institute reforms such as a shift to preventive care delivered by the PHC network was also a more difficult task in these geographic areas where there existed few options for curative care, thus reducing the capacity to refer to a close higher echelon of care [9].

Sixth, the past shocks resulted in a sometimes unstable and insecure pathway of access for all the patients who could not afford the private and for-profit system. For poor Lebanese, the prevailing pattern is a minimal government provision of primary healthcare through a discounted rate compensated by the MoPH through technical and in-kind support. Lebanese employed in the formal sector benefit from a combination of national funds and expensive private care [2, 8]. Palestinian refugees are supported by UNRWA, an agency that has been struggling to maintain funding over time. For Syrian refugees, access to primary healthcare is partly subsidized through a preferential rate compensated by financial support from the UNHCR [4, 9]. However, the support capacity of both UNHCR and UNRWA is facing constraints and funding gaps [10], which might be worsened in the future by the financial burden that the Covid19 pandemic is imposing on the economy of donor countries.

### Resilience mechanisms to work long

Throughout the past decades of unrest, the MoPH PHC department supported by a range of local and international actors created a space for collaboration beyond the sectarian lines and across the geographic disparities inherited from the past. This amplitude for delivering public primary healthcare services, expanding beyond sectarian, gender, religious, geographic or political divides, is referred to in this paper as the “space for public health as a public good” in Lebanon. The creation of such a space was achieved by ensuring a role for the MoPH to coordinate an agreed-upon set of national frames and standards for all autonomous PHC facilities adhering to the network and was informed by a team of grounded national and international actors believing in public health principles [2]. Based on these principles, the MoPH engaged to strengthen the capacity of the PHC centres within the network through technical tools such as trainings and guidelines, access to the PHCN health information system, the inclusion into an accreditation scheme and access to subsidies and in kind supports. In return, PHC centres committed to provide the basic services required by the MoPH at discounted rate; to adhere to national health, safety and clinical regulations and standards; and to provide regular reports to the MoPH. The national Primary Healthcare Network (PHCN) was established in 1996 with 19 primary healthcare centres adhering to the network [7, 9]. Today the network includes 237 PHC centres distributed across Lebanon, serving over 1 million beneficiaries annually including poor Lebanese and Syrian and some Iraqi and Palestinian refugee populations.

To make it possible for the over one million Syrian refugees to be hosted in Lebanon, the international community raised additional substantial resources which were funnelled to the national government [9, 10]. Key to this effort was the intent to reduce the tension between and among populations and to support the delivery of healthcare services in impoverished areas [4, 9, 10]. In terms of the PHC centres, some very important clinical and administrative supports were provided to mitigate the crisis effects on the most impoverished Lebanese. These were funded by the European Union and the World Bank to include proactively the vulnerable Lebanese in the response to the crisis and structured to build a longer term program with a package of subsidized care for PHC services for all population groups [9].

Today the PHC network represents around one quarter of all facilities providing outpatient care in Lebanon. As a growing number of actors have been joining under the MoPH leadership, a collective non-profit provision of primary healthcare has been forged. Despite its lack of direct capacity, the MoPH PHC team has shaped convening mechanisms that aim at a fairer distribution of resources which takes into account the essential needs of the most vulnerable population groups described above. The creation of a cohesive monitoring process also supports the establishment of an information platform where the data collected routinely can be analysed and presented. This information platform creates a concrete public good and also embodies a way to maintain an understanding of the reality and reach and gaps of the public health enterprise.

Several difficult systemic issues in the PHC network remain, however. These include the complexities of the referral processes; recalcitrant gaps in matching professional services with patient needs at different primary healthcare centres; an overall high level of staff turnover; and challenges posed in trying to manage the drug supply chains in the network [7, 9]. These persistent difficulties show that the national and international efforts deployed have not been sufficient to decrease inequalities and ensure access to basic health services for all the most vulnerable and that the resilience mechanisms deployed remain fragile [4]. The cumulative effects of the division along sectarian lines, the privatization of healthcare, the lack of legal mandate and supervisory authority to guide healthcare provision, and the very low resources channelled towards ensuring field supervision and quality control have continuously undermined the aspiration to make primary healthcare a common public good. Yet the efforts deployed by the PHCN to build a people-centred approach at the level of primary care [2, 4, 9] have persisted and the weight of the past and current crises has not extinguished the vision of Universal Health coverage at the core of the MoPH PHC team strategy [4].

### The shocks of the present

In this section we consider how the fissures of the past have undermined capacities to withstand the blows of the present, as two unprecedented shocks battered the country in less than a year apart. The first shock hit Lebanon in October 2019 when the economic crisis developed into a full-blown economic collapse [13]. Loss of confidence in the local currency led to effective devaluation of the market rate for the Lebanese Lira and resulted in a debilitating financial crisis with a shortage of stable foreign currencies within Lebanon’s economy [13]. Unemployment rates skyrocketed as hundreds of businesses closed down [3]. These developments in turn created shortages in basic supplies and inflation in prices of goods. Economic experts say Lebanon will experience a deep recession and a double-digit contraction in the economy equivalent to what the United States experienced during the Great Depression [13]. The second shock, in a country increasingly oppressed by a crumbling economy, was the blast that blew apart the Beirut port on August 4, 2020. On that day, 2′750 tons of ammonium nitrate left “lying around unsafely for the better part of a decade” [3] exploded with ruinous force. The blast that struck the heart of the country is said to be equivalent to 1/10 of the blast effects of the atomic bomb dropped on Hiroshima. The powerful explosion led to at least 180 deaths and an estimated 6000 people injured [14]. Over 110 people remain missing and hundreds of thousands of people have been left homeless in the capital city.

We document below the cumulative effects of these two brutal present shocks. We describe their dreadful impacts on the MoPH readiness, on the population capacity to adapt and on the longer-term dependencies that combined might undermine the decades-long efforts to protect primary healthcare as a public good in Lebanon.

#### Restrained capacities to work short

At the structural level, these two shocks have constrained further the capacity of the MoPH. The primary healthcare sector has been badly battered by the economic crisis on multiple levels. The economic collapse has markedly reduced the ability to import essential drugs, medical supplies, and equipment [2]. Access to basic services such as water and electricity has been diminished due to shortages in gasoline and diesel resulting from the inflation, the shortage of stable foreign currencies and the consequent inability to import from abroad. Hospitals and healthcare facilities are unable to operate at full capacity and are using their scarce resources to maintain utilities (in terms of water and electricity) while accepting only patients in need of emergency care and delaying routine operations.

Domestic funding for this sector has also been either frozen or rescinded. Existing budgets allocated to the national PHC network for procurement of vaccines, chronic and essential drugs, and reproductive health supplies are not being transferred or disbursed by the Ministry of Finance due to lack of available funds. The PHC department at the MoPH itself has not received funding--and this has been the case for almost two years. This absence of regular funding is leading to stock-outs in medication at the level of the PHC centres. In order to prevent discontinuation of medication at the level of the beneficiaries, the government has temporarily shifted the burden of procuring the medicine to the PHC centres and their related NGOs, for what is termed “the interim stock-out period.” In addition, operational costs for the PHC network are increasing while resources are dwindling, particularly the electricity costs at the central drug warehouse (essential to maintaining the cold chain) and the transportation costs to conduct the quality monitoring field visits. As for human resources at the PHC department, funding has almost disappeared with the existing team now only one third of what it was two years ago. This reduction in staff leads to decreased ability to conduct quality follow-up within an increasingly more stressed PHC network. At the level of the PHC centres, operational costs have also drastically gone up. The PHC centres are having to pay more in terms of (i) supplies which are all imported on the dollar rate and (ii) generators and back-up electricity required to compensate for daily long outages. In addition, revenues have gone down as poverty rates increase and beneficiaries can no longer afford the already subsidized fees.

As a result, the PHC centres are resorting to measures to decrease their operational costs in other ways such as (i) giving half salaries or turning full time positions into part time; (ii) stopping imports of expensive supplies; and (iii) switching to more affordable supplies like chlorine for sterilization instead of other disinfectants. If PHCs are not able to procure the medical supplies by themselves, patients are forced to undergo months of service discontinuation. Qualified and unpaid health staff are currently emigrating abroad to seek better opportunities. PHC centres and NGOs are requesting an increase in the consultation fee at the PHC network to match the 40% increase in payment to the private sector as announced by the Order of Physicians. This request has been met with stark rejection at the level of the MoPH which is keen on maintaining low fees and securing the access of the vulnerable population to these essential services. It is expected in the near future that some PHC centres, particularly those in rural areas or run by small NGOs, might close. Other large NGO-supported health centres might merge with PHC centres to reduce costs.

In less than one year after the full economic collapse, the unprecedented explosion in Beirut added another deep blow to the MoPH service capacity [3]. Following the blast, three hospitals, twelve primary healthcare centres and the MoPH main warehouse have been severely damaged. When hospitals were not rendered partially or completely dysfunctional by the blast and fires, they were rapidly overwhelmed by injured people. The blast has destroyed five out of seven UNICEF-supported vaccine cold rooms. Members of the MoPH team writing this article tried to salvage 90% of the stock in the 72 h following the disaster, with the support of UNICEF. The violent destruction of these core assets contributes to further disable the health system, already crippled by the shortages in fuel, electricity, medical supplies, and water which for months have been affecting both the private and the public systems [2].

#### An increased number of people to support while working short

Some projections of the effects of the economic collapse estimate that poverty rates could rise to include more than 45% percent of the Lebanese population, leaving 1.6 million people unable to afford food and basic non-food items [2, 13]. Some estimations mention that by the end of 2020 poverty rates among Lebanese could reach 75% of the population [3].

The drastic reduction of purchasing power as a result of higher unemployment and rising prices has already led to major difficulties in maintaining a minimum livelihood [3]. As people struggle to make ends meet and to procure basic food items, accessing healthcare services has become a luxury and an increasingly larger proportion of the population is priced out of even the subsidized health care services. As the proportion of impoverished Lebanese is rising, the populations at the edge of poverty are more likely to neglect preventive care or to try to self-manage their chronic diseases, since clinic outpatient care is not covered at the point of care by most private insurance schemes and is reimbursed subsequently by the national security fund. As a result, and in order to avoid spending money for preventive measures, people delay seeking care until their condition is critical and then they bypass the primary level to present directly to hospitals where secondary care can be covered by the MoPH for the most vulnerable Lebanese or by the national fund if people are employed [2, 4]. Yet in current circumstances, as unemployment rises and Lebanese families inevitably lose their associated health insurance, the MoPH is now responsible for covering the care of a much larger proportion of the population who are presenting in an increasingly critical state. These current circumstances reinforce the geographic disparities and drive ever increasing complex and unstable pathways to access care.

#### An increasing dependency on external supports while working short

As an emergency response to mitigate some effects of the economic breakdown, the MoPH is working with a range of external actors. These include the EU- Madad initiative (set up by the EU in response to the Syrian crisis in the region) as well as UNICEF, UNFPA, WHO, and UNHCR. These actors collaborate to fund the procurement of the vaccines and drugs for the national PHC network, which benefits both Syrian refugees and the vulnerable host Lebanese population. These actors are also playing a major role in the COVID-19 response through procuring Personal Protective Equipment (PPE) for the PHC network response. The Beirut port explosion also destroyed ten containers of PPE, which remain essential for the national Covid-19 response. In response to the Beirut blast, the MoPH along with national and international partners developed the immediate relief model (IRM) which aimed at subsidizing care for the beneficiaries of the 21 PHC centres that were directly affected by the blast. Although the international agencies are stepping up to fill these gaps, these emergency measures create further dependency in Lebanon on external funding in the public healthcare sector. This enforced dependency may have spill-over effects in shaping the funding, the role, and the coherence of the future primary healthcare response in Lebanon.

### Covid-19 and the sharp drop in utilization observed in the PHCN

**Fig. 1:**
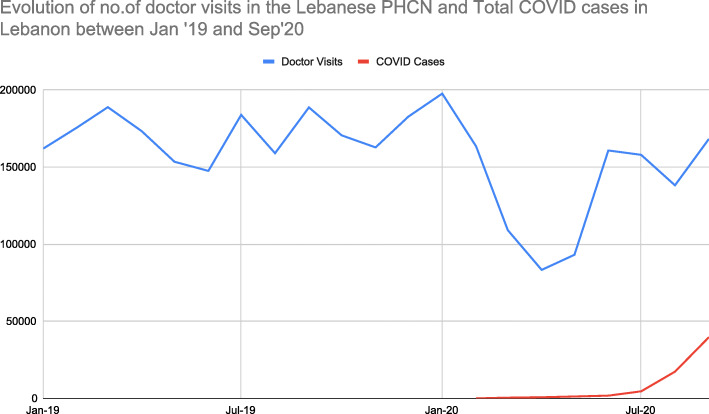
Number of visits to doctor monitored between January 2019 and September 2020

At the outset of the COVID-19 epidemic in Lebanon, the MoPH PHC team began to notice from the routine PHCN monitoring data a decrease in patient visits for essential services. An analysis of the evolution of the utilisation rates per type of consultation was performed between January 2109 and September 2020, as the Covid-19 pandemic increased in intensity (Fig. 1). The overall observation is that visits for essential services during the strict lockdown period have declined by up to 70%, depending upon category.

Socially, economically, and politically, the situation has now become much more complex since the first case of Covid-19 was identified in Lebanon on 21 February 2020 [2]. The first complexity is a higher risk for Lebanese services to become fragmented along sectarian lines in the midst of the political turmoil and related social tensions exacerbated by these shocks. The ongoing unrest could trigger a massive withdrawal from the weakened mechanisms for a coordinated management of the multi-layered crisis and could lead to political parties turning back to historic fault lines. Such a fragmentation would further constrain the possibilities to develop cohesive response measures and would limit the capacity to understand by the agency of the multiple components of the Lebanese health system, how Covid-19 is spreading throughout the population. Such a fragmented approach might also create pockets of populations left unattended as the prior coordinated patterns of information and access become disrupted. The pandemic trends in Lebanon suggest that ascertainment issues (testing, contract tracing, isolation) may be insufficient to contain the spread of the virus. The efforts of the health system today may converge towards taking care of the injured from the blasts and the political unrest, re-constituting the hospital infrastructure and ensuring treatment for severe Covid-19 patients. The Intensive Care Unit (ICU) beds in the private system remain unaffordable for poor populations and therefore are to be covered by the MoPH.

Another crucial constraint on anything but a short-term perspective is the utter absence of effective leadership and the reciprocal complete lack of citizen trust in the capacity of the government. The population as a whole, without an adequate spokesperson, might have difficulty in clarifying to the humanitarian agencies, coming in to act in an emergency mode to contain the pandemic, the urgent need to protect primary healthcare and preventive services and to protect public health as a public good. Social distancing measures might be more difficult to implement among populations living in poor and overcrowded environments, while the economic effects of confinement measures will most heavily affect the people vulnerable economically and socially. The pandemic has differentially affected previously impoverished areas including central Lebanon (encompassing the city of Beirut) as well the North, the South, and the Beqaa. The cases detected seem to have been occurring in three successive waves, with a sharp increase in the second half of 2020. An additional fundamental tension is that the response to Covid-19 has made claims on the material and human capacities of the health system in terms of protective equipment, drugs, expertise, assessment, triage, referrals, quarantine, inpatient treatment, and coordination. But the health system today has much less capacity than before the recent shocks, given the blows it has suffered as discussed above.

### Four essential questions to work short and long

Based on its historical mission, its current intent and the challenges posed by the Covid-19 pandemic, the PHCN response strategy must ensure that the network can meet the most urgent pandemic needs while maintaining a coherent longer-term approach to support its historical resilience mechanisms and protect the space for primary healthcare as a public good in Lebanon. These mechanisms include access to essential services for the most affected, including now the people who have lost everything in the economic collapse and in the explosions in Beirut.

In order to work both short and long in a coherent way, it is necessary to understand what role the PHCN might play to support the Covid-19 response while maintaining essential service functions for an increasing number of vulnerable population groups, when hospitals are now overwhelmed. It is necessary to frame the challenges posed by Covid-19 in the light of these inherited difficulties, because in this framing, with insights from the past and from the present, it is possible for the PHC community to figure out how the trade-offs imposed by these combined shocks are negotiated at the intersection between the people affected and the capacities of the PHCN system. It is also crucial for international and national actors engaged in making policies or framing a response to understand whether such trade-offs are addressed in ways that maintain the capacity and coordination role of the MoPH and meet the needs of the population to be covered. The empirical context described above would suggest four sets of questions pivotal to the PHC community in framing the appropriate field response.

The first set of questions relates to the health system’s capacity to detect, trace, refer and protect the population from the specific risks that are posed by Covid-19 in a complex and fragmented PHC network, which is facing serious limitations to manage the response in a comprehensive and systematic way. Seasoned assessments from the health sector are needed to understand what is currently feasible in terms of identification, referrals, and coordination in the current PHC network, in order to avoid a further increase of Covid-19 cases.

The second set of questions relates to the capacities of facilities in the network to deal with key gaps -- in human resources, laboratory capacities, supply chains and essential stocks of medications and materials -- and whether it is justified from an ethical standpoint in such a situation to shift these scarce resources towards a Covid-19 response at the level of the PHCN.

The third set of questions relates to the population and the need to understand whether their most essential primary healthcare needs can be met while focusing on a Covid-19 response at the level of the PHC network. This concern is crucial especially during the extended effort it will now take to mount an adequate disaster response and meet the health and mental health needs of the injured survivors, their families and the people crushed by the economic breakdown. These issues require understanding the health and social impacts of not meeting essential needs and the potential for social breakdown that such decisions might entail in the longer run. This third set of questions devolves into one main one: Is failing to meet several essential needs in a trade-off made to respond to Covid-19 actually an option in the fragile social and disaster-stunned context of Lebanon today?

The fourth set of questions relates to how the international supports to the economic collapse and the port disaster are organized and channelled. The key question here is whether such emergency supports take into account the broader picture: The need to prevent the spread of Covid-19 cases as well as to ensure essential access to services at the level of PHCs for all populations trapped in this protracted conflict in the short and long run.

## Conclusion

Globally, Covid-19 has pushed health systems to their limits [15]. In resource -constrained contexts where the burden of disease and risk factors are often distributed unevenly in the population, factors such as siloed financing and fragmented governance of the health system may well have contributed to an increased burden on the poor [15]. Today, service providers and policy-makers worldwide are pressured to decide which essential services must be protected as precious resources are reallocated [16]. Making sound decisions is even more difficult in a context where political, economic or financial interests might well result in fragmented directives that privilege the powerful and thus serve to increase inequities [15].

Public health decisions and responses are anchored in principles including the notion of justice and equity [17]. It has been recognized that around the world the poor are experiencing more severe direct and indirect consequences of Covid-19. To disregard the short- and long-term effects of the pandemic may have costly impacts on the society overall, as Covid-19 is affecting both the demand for and the provision of essential services [15–17]. A one size fits all response is likely to cause great hardship for those most in need [17]. In contexts where constrained resources are now further dwindling, it is crucial to understand the context of the pandemic, the capacities of the health systems, and the needs of the community. In such settings, the decisions made to control the Covid-19 pandemic could impose even more drastic and inequitable short- and long-term consequences on health systems and on populations affected [15–17].

In the case of Lebanon, important steps have been initiated in the past two decades to improve governance, increase the financial support and reform the service delivery at the level of the public PHC network [9]. Today, the local longer-term priorities might be ignored by the international aid community in an attempt to avoid reliance on the untrusted governmental structures [3]. Yet a humanitarian response that does not protect the efforts and the results reached over the past decades would fail to preserve this hard-won public health space for the most vulnerable populations. In Lebanon today, the principles of public health might serve as the grounds on which to build solidarity [15]. It is in these situations where values are challenged that it is essential to ensure that the aid responses are able to recognize the need to include everybody while working short and long, along the fissures left by the past and navigating within what needs to be done today.

All these issues collide because Lebanon is deeply in the midst of a protracted conflict and faces a pandemic. The set of questions proposed are being incorporated in an empirical field study to be conducted in collaboration with field response actors. The answers to these questions will allow the MoPH PHC team to understand whether, in the context of Lebanon, continued access to essential healthcare proves to be pivotal for people as they suffer from Covid-19; and whether that access is valued in part because it sustains among the people a sense of social security and continuity. These questions link directly to how the PHCN might be able assert its role as a guardian of the fragile space in which primary healthcare will be maintained as a public good in Lebanon not just for now but for tomorrow.

## Data Availability

All articles used as reference were available in open source.

## Abbreviations

EU-Madad: European Union Regional Trust Fund in Response to the Syrian Crisis
ICRC: International Committee of the Red Cross
ICU: Intensive Care Unit
MoPH: Ministry of Public Health
NGO: Non-Governmental Organization
OOP: Out-of-Pocket
PHC: Primary Health Care
PHCN: Primary Health Care Network
PPE: Personal Protective Equipment
UN: United Nations
UNFPA: United Nations Populations Fund
UNHCR: Office of the United Nations High Commissioner for Refugees
UNRWA: United Nations Works and Reliefs Agency for Palestinian Refugees
US: United States
WHO: World Health Organization
